# Quantitation of *α*-Lactalbumin by Liquid Chromatography Tandem Mass Spectrometry in Medicinal Adjuvant Lactose

**DOI:** 10.1155/2014/841084

**Published:** 2014-12-04

**Authors:** Rui Yan, Longmei Qu, Nan Luo, Yang Liu, Yu Liu, Li Li, Lijiang Chen

**Affiliations:** ^1^College of Pharmacy, Liaoning University, Shenyang 110036, China; ^2^Yichang Humanwell Pharmaceutical Co., Ltd., Yichang 443005, China

## Abstract

Lactose is a widely used pharmaceutical excipient, sometimes irreplaceable. Traces of residual proteins left during production of lactose are potential allergen to body. The present paper describes a sensitive and specific LC-MS method for the determination of *α*-lactalbumin (*α*-La) in lactose samples. Chromatographic separation was performed on an Acquity UPLC BEH300 C18 column (2.1 × 150 mm, 1.7 *μ*m) with an isocratic mobile phase consisting of water containing 0.1% TFA and acetonitrile containing 0.1% TFA (80 : 20, v/v). Mass spectrometric detection was achieved by a triple-quadrupole mass spectrometer equipped with an ESI interface operating in positive ionization mode. Quantitation was performed using selected ion monitoring of *m/z* 2364 for *α*-La. The calibration curve was linear from 0.2 to 10 *µ*g/mL. The intra- and interday precisions were less than 7.6% and the accuracy ranged from 96.4 to 104.5%. The limit of quantification (LOQ) was 0.15 *µ*g/mL and the limit of detection (LOD) was 0.05 *µ*g/mL. This method was then successfully applied to investigate 6 different lactose samples. The application can provide technical preparation for the development of specification of lactose.

## 1. Introduction

Lactose is commonly used as an inactive ingredient in many pharmaceutical formulations including tablets, suspensions, dry powder inhalers, and medicines for injection. Since lactose is manufactured from bovine whey, through degreasing, protein removing, concentrating, crystallizing, and drying, residual whey protein is inevitable in lactose products. Recently, allergy reactions of injections containing lactose in clinical practice were reported, where the allergen was residual whey proteins [1]. A previous report described a cow's milk allergy in an asthmatic patient who had an anaphylactic reaction to the milk protein in a dry powder inhaler containing both salmeterol and fluticasone [2]. In addition, residual proteins can provide nutrient components to stimulate the growth of viruses so that lactose may carry some viruses. The residual proteins include *α*-lactalbumin (*α*-La), *β*-lactoglobulin (*β*-Lg), bovine serum albumin (BSA), immunoglobulin (Ig), and other albumins. “China biological products procedures” only include the stipulation that the content of residual BSA should not exceed 50 ng/mL per person. However, Pharmacopoeia of China or other countries record no relevant quality specifications on lactose for injection so far. It is urgent to develop determination methods for residual protein in lactose for injection to investigate the quality of the pharmaceutical adjuvant.

Traditional methods for proteins determination include Kjeldahl method, Lowry method, and Bradford method. In recent years, some publications have described methods utilizing IR [3], CE [4], amino acid analysis [5], and spectrophotometry [6, 7] to quantify proteins in complex matrices. Yang et al. reported a modified Kjeldahl method for the quantification of residual protein in lactose for injection [8]. More and more experimental applications prove that LC-MS is a powerful determination tool for proteins due to the versatility, high resolution, and short analysis time of the method [9–11]. As one of the most important allergens in lactose, *α*-La was determined in medicinal lactose by a capillary zone electrophoresis (CZE) method with a LOD of 3.0 *μ*g/mL [12]. In the present study, a suitable LC-MS method for determination of *α*-La in lactose for injection was established. Application of the method to commercial lactose samples could provide references for relevant drug administration to set limits of residual *α*-La for lactose for injection.

## 2. Experimental

### 2.1. Materials

*α*-Lactalbumin (purity ≥ 85%) protein standards were purchased from Shanghai Yuanye Bio-Technology Co., Ltd. (Shanghai, China). Acetonitrile and methanol were of HPLC grade (Sigma-Aldrich, St. Louis, MO, USA). Acetic acid, formic acid, trifluoroacetic acid (TFA), and other solvents were of analytical grade. High quality poly(9,9-diethylfluorene) (PDEF) syringe filters (0.22 *μ*m pore size; 13 mm diameter) were purchased from Sigma-Aldrich. Watsons pure distilled water was used (A.S. Watson Group Ltd., Hong Kong).

### 2.2. Instrumentation

The LC-MS/MS system consisted of an Agilent 1290 series liquid chromatograph and an Agilent 6460 triple-quadrupole mass spectrometer (Agilent Technologies, Inc., USA) with electrospray ionization. Data acquisition and processing were processed using the Agilent MassHunter Chemstation (B.01.03).

Separation of peptides was performed on a narrow-bore Acquity UPLC BEH300 C18 column (2.1 × 150 mm, 1.7 *μ*m) from Waters (Milford, MA, USA), maintained at 30°C. The mobile phase consisted of water containing 0.1% TFA and acetonitrile containing 0.1% TFA (80 : 20, v/v) with a flow rate of 0.1 mL/min, and the injection volume was 10 *μ*L.

The Dual AJS electrospray ion source was operated in the positive ion mode (the gas temperature, 350°C; drying gas, 13 L/min; nebulizer, 45 psig; sheath gas temperature, 250°C; sheath gas flow, 11 L/min; VCap, 3500 V; nozzle voltage, 1000 V; fragmentor, 320 V). Quantitation was performed under the selected ion monitoring mode (SIM) of *m*/*z* 2364.

### 2.3. Preparation of Blank Matrix

0.2 g of lactose (batch number: K43367545237) was weighted accurately and dissolved in 25 mL water. The solution passed through a 0.22 *μ*m filter and was ready for injection.

### 2.4. Preparation of Standard Solutions

The stock solutions (1 mg/mL) of bovine *α*-La were prepared in water. A mixed stock solution containing 100 *μ*g/mL of bovine *α*-La was prepared in the blank matrix. All stock solutions were stored in the dark at −20°C for no more than 1 month. Work standard solutions were prepared from these stock solutions and were diluted step by step with the blank matrix immediately before using.

### 2.5. Sample Preparation

0.2 g of lactose sample was weighted accurately and dissolved in 25 mL water. The solution passed through a 0.22 *μ*m filter and was ready for injection.

## 3. Results and Discussion

### 3.1. Optimization of LC-MS/MS for Quantitative Analysis

**Figure 1:**
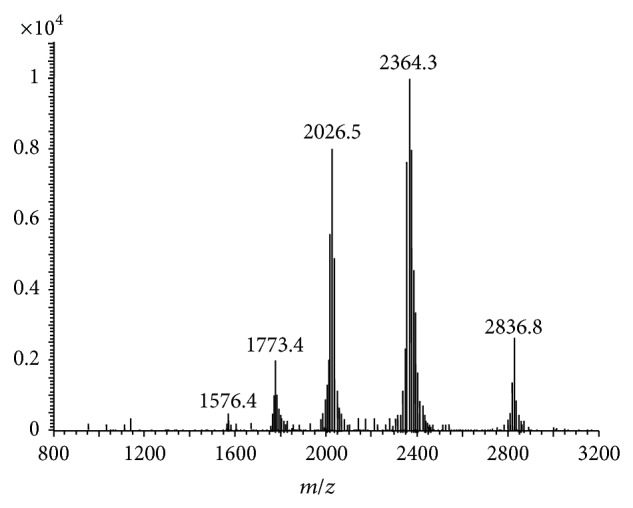
The original mass spectra of bovine *α*-La.

The full-scan spectra of the bovine *α*-La are shown in Figure 1; peak of 6+ (*m*/*z*, 2364) charge state was chosen because of the high relative abundance. To obtain maximum sensitivity of the SIM, some parameters such as spray voltage, capillary temperature, sheath gas flow, and nebulizer pressure were optimized. The other MS parameters were adopted from the recommended values for the instrument.

The optimization of chromatographic conditions was mainly guided by the requirement for quantifying assay and reducing the analytical run time. Different compositions of mobile phase were compared for their separation efficiency. The results showed that TFA is indispensable for obtaining good chromatographic peaks that the selected mobile phase as Section 2.2 could provide specificity and proper retention time. Each chromatographic run was completed within 5 min.

### 3.2. Analytical Performance

To validate the LC-MS method for determination of bovine *α*-La in lactose, the analytical performance of the method should be satisfactory with the linearity, sensitivity, recovery, precision, and repeatability.

#### 3.2.1. Linearity and Sensitivity

The calibration curve was established after injection of the standard solutions in duplicate at six concentrations. *α*-La standard solution, with the concentration sequence of 0.2, 0.4, 1.0, 2.0, 4.0, and 10.0 *μ*g/mL, was prepared in mobile phase. All calibration curves were linear over the range of 0.2–10.0 *μ*g/mL with correlation coefficients (*R* ^2^) ≥ 0.9991.

The limit of detection (LOD) and limit of quantitation (LOQ) were the concentrations of a compound at which its signal-to-noise ratios were detected as 3 : 1 and 10 : 1, respectively. They were determined by serial dilution of sample solutions under the described LC-MS conditions. The LOD and LOQ of *α*-La were 0.05 and 0.15 *μ*g/mL, respectively.

#### 3.2.2. Recovery and Precision

Recovery was performed employing the method of standard addition. Low, intermediate, and high levels of *α*-La standards were added to approximately 0.1 g of sample, the content of which had been determined. The sample was then processed and analyzed as described in Section 2.5. Three parallel samples were prepared and the assay was repeated three times. The results are summarized in Table 1. All recoveries were in the range of 96.4–104.5%.

The precision of the method was evaluated by six replicate analyses of the same lactose sample over consecutive three days. Intra- and interday precision at low, intermediate, and high concentration levels are listed in Table 1. The RSDs were in the range of 1.8–4.7% for intraday precision and 2.3–7.6% for interday precision.

#### 3.2.3. Repeatability

The repeatability of the LC-MS method was studied by determining the variation in relative MS response when analyzing the same lactose with 6 sample preparations. The RSDs were ≤5.7%.

#### 3.2.4. Stability

The stability of the analytes in the prepared samples stored at room temperature was investigated by replicate injection of the sample solution at 0, 2, 4, 8, and 12 h. The RSDs of the assay results at different time intervals were 1.6–3.5%, which indicated that the sample solution was stable at room temperature for at least 12 h.

### 3.3. Method Application

**Figure 2:**
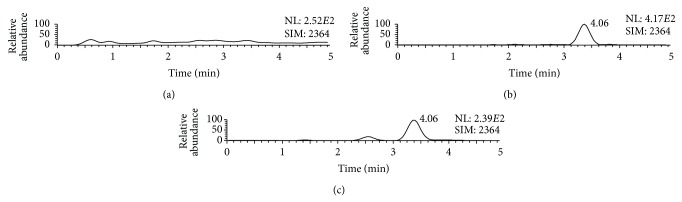
Representative SIM chromatograms for *α*-La of (a) water, (b) water spiked with *α*-La at the LLOQ, and (c) a lactose sample.

The validated analytical method was applied to 6 lactose samples from different sources. A representative SIM chromatogram of the lactose sample is shown in Figure 2. The quantitative results were summarized in Table 2.

## 4. Conclusion

A sensitive and selective LC-MS method was developed for the determination of bovine *α*-lactalbumin in pharmaceutical adjuvant lactose. The method has the LOQ of 0.15 *μ*g/mL and allows accurate quantification of low levels of *α*-La. It was successfully applied to determine bovine *α*-La in six different lactose samples. This paper proved that it is feasible to assay residual proteins in lactose using LC-MS and also prepared for the development of quality control of lactose for injection and other pharmaceutical adjuvants containing *α*-La. Its application might be helpful to pharmaceutical enterprise on new drug registration.

## Figures and Tables

**Table 1:** Recovery and intra- and interday precision of LC-MS method (*n* = 3).

Nominal (*μ*g/mL)	Recovery (%)		Precision (%)	
	Mean	RSD%	Intraday	Interday
0.4	104.5	6.3	3.1	5.5
2	96.4	2.9	4.7	7.6
8	98.6	3.6	1.8	2.3

**Table 2:** Detected contents of *α*-La (*μ*g/g) in six commercial lactose samples from different sources.

Batch number	K43367545237	11008	L1139	10670473	10691931	10652704

Mass fractions (*μ*g/g)	Not detected	52.7	78.4	46.3	39.5	54.3
